# Recycled Thermoplastics for 3D Printing Filament Production: A Review of Circular Economy Drivers, Material Behavior, and Current Research Gaps

**DOI:** 10.3390/polym18101174

**Published:** 2026-05-10

**Authors:** Zuzana Mitaľová, Jakub Kaščak, Marek Kočiško, Juliána Litecká

**Affiliations:** 1 Faculty of Manufacturing Technologies, Technical University of Kosice, Bayerova 1, 080 01 Presov, Slovakia; jakub.kascak@tuke.sk; 2 Faculty of Humanities and Natural Science, University of Presov, 080 01 Presov, Slovakia; juliana.litecka@unipo.sk

**Keywords:** filament, recycling, sustainability, additive technology

## Abstract

The article focuses on the potential of recycled materials for the production of 3D printing filaments.. The individual parts are focused on defines the relationship between circular economy/3D printing technology, and the key motivations for the use of recyclates in the context of sustainability. Core of the article describes different types of recycled polymers, with emphasis on the number of recycling cycles and the associated changes in material properties. It also includes a discussion of degradation processes resulting from repeated thermal loading, i.e., mechanical recycling. Simultaneously, individual recyclates are comparatively evaluated in terms of mechanical properties, rheological characteristics (particularly the melt flow index), and their processability in 3D printing. Furthermore, key challenges are identified, and perspective directions for future research in this field are outlined.

## 1. Introduction

**Figure 1:**
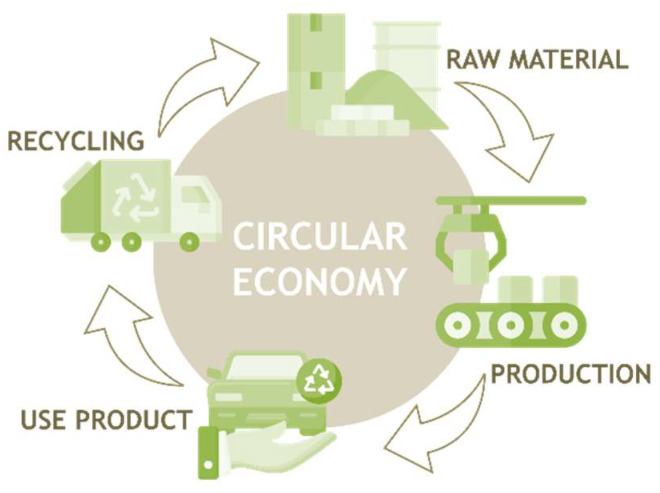
Circular economy model—counterclockwise direction (authors’ own processing).

The objective of the circular economy is to design products/materials in such a way as to enable their repeated use with the smallest possible loss of value and without generating harmful emissions into the environment. One of the primary reasons for introducing the circular economy (CE) model into manufacturing is the disproportionate increase in demand for raw materials, which rises in parallel with global population growth (in the case of European Union countries, a crucial factor is also their dependence on imports of raw materials from third countries). The idea of the circular economy (Figure 1) represents the opposite of the linear model developed in the period following the Second World War. The linear model gradually entered into crisis due to its incompatibility with the principles of sustainable development, since its “output” was non-recyclable waste. The CE model is not inherently innovative; several documents indicate its origins in China. However, the concept was explicitly elaborated only in 1989 (Pearce and Turner). The essence of CE is aptly captured by Stahel, who describes it using the term “lake economy” as an antithesis to the continuous flow of the “river” in a linear economy. The principles of the circular economy include the 3Rs or 6Rs (Reduce–Reuse–Recycle–Recover–Remanufacture–Redesign). The benefits of implementing CE principles include environmental and climate protection, reduced dependence on primary raw materials, biodiversity restoration, waste minimization (with the aim of eliminating waste altogether), and support for economic growth and enterprise competitiveness [1,2,3,4,5,6,7,8].

The relationship between CE and 3D printing technology (additive manufacturing) can be understood as an interaction between a macroeconomic concept and a manufacturing technology. CE represents a systemic approach, whereas 3D printing serves as a “tool” that supports this approach in the following areas:

- Extension of product life cycles—on-demand production of spare parts eliminating the need for inventory storage);
- (Topological) design/lightweighting → reduction in material consumption;
- Reduction of transport-related emissions—enabling localized production closer to the end user/decentralization of manufacturing;
- Utilization of recycled materials as feedstock for 3D printing (closed-loop systems).

In the last-mentioned area, namely the application of recyclates in 3D printing, the primary limiting factor is the progressive degradation of material properties during the recycling process.

The article is structured into four principal sections. Section 2 briefly describes the key motivations for the use of recyclates in the context of sustainability. The subsequent Section 3 describes the types of recycled polymer materials suitable for the production of 3D printing filaments, with a focus on their properties after repeated thermal loading. In Section 4, individual recyclates are comparatively evaluated, and key challenges are identified.

## 2. Secondary Raw Materials for 3D Printing in the Context of Sustainability

The valorization of waste materials reprocessed into materials for a new purpose is gaining importance within additive manufacturing technologies. With the development of recycling processes and in line with the European Union (EU) action plan (aimed at creating a market for secondary raw materials by 2026), recycled filaments for 3D printing are being produced. Virgin materials for filament production require a high degree of dependence on petroleum-based products (up to 2.5 tons of oil are consumed to produce 1 ton of plastics). This is associated with so-called unsustainability, as resources are ultimately depleted, as well as with a high carbon footprint in the production/disposal process, especially via incineration. As a result, the recycling and reuse of polymers emerge as key strategies for reducing environmental burden and enabling the transition toward a more sustainable materials economy. The global production of various types of plastics over the past five years is presented in Table 1. However, only approximately 10% of plastics derived from fossil resources are mechanically recycled.

**Figure 2:**
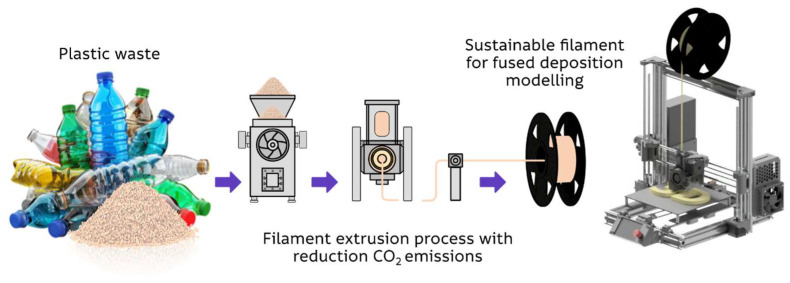
Filament production from waste (graphical representation).

Mechanical recycling of plastic waste for 3D printing filament production (graphically represented in Figure 2) is addressed, for example, by ProjectSeafood, Plastic Bank, Perpetual Plastic Project, etc. A significant reduction in carbon footprint associated with the use of recycled materials is reported by manufacturers; for instance, companies such as Prusa and Kimya declare CO_2_ emission reductions of approximately 35–57%. However, it should be emphasized that these values represent commercial estimates [1,10,11,12,13].

Material recycling is defined as the repeated thermal processing of waste. This approach is considered suitable primarily for waste streams based on thermoplastic polymers. It comprises a sequence of processing steps, including collection, sorting (according to recycling code marking: 1 PET—Polyethylene terephthalate, 2 HDPE—High-density polyethylene, 3 PVC—Polyvinyl chloride, 4 LDPE—Low-density polyethylene, 5 PP—Polypropylene, 6 PS—Polystyrene; codes 7–19, e.g., PC—Polycarbonate, ABS—Acrylonitrile butadiene styrene), mechanical size reduction to achieve the required fraction size, the washing/drying phase, and regranulation [14,15].

Recycled materials are denoted in the text by the prefix r placed before the specific polymer abbreviation (e.g., rABS—recycled ABS). Virgin materials are denoted by the prefix v (e.g., vABS—virgin ABS).

## 3. Recycled Polymers for 3D Printing

### 3.1. Recyclate Acrylonitrile Butadiene Styrene

ABS in its virgin form is one of the most widely used (universal) materials for filament production. It has good processability and a low melting temperature (approx. 225 °C). The material is resistant to high mechanical loading and temperature (suitable for spare machine parts exposed to sunlight/hot water flow), strong, and slightly flexible. Final surface finishing of ABS parts may be performed by sanding (easier than for PLA) or by acetone application to achieve surface gloss [16,17].

A broad spectrum of recyclate properties is evaluated in study [18]. TGA analysis of recyclates shows no major changes compared with virgin ABS. The surface of the recyclate material is more homogeneous than that of the virgin form. The recyclate can also be prepared as a blend, with a 1:4 ratio (vABS/rABS) considered optimal. The extrusion temperature for the reported blend ranges between 190 and 195 °C; at higher temperatures, thermal degradation occurs. With respect to the number of recycling cycles, the authors of [19] report the possibility of repeated mechanical recycling up to 5 times without a significant effect on mechanical property characteristics.

**Figure 3:**
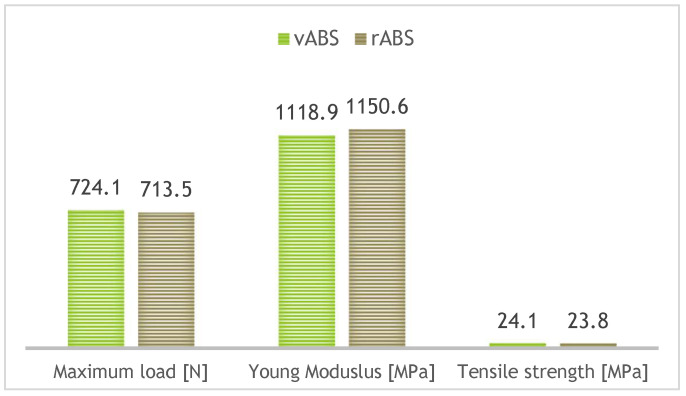
Selected mechanical property characteristics of vABS/rABS [19].

During recycling, nanoparticles and fumes are released into laboratory spaces, which represents a health risk. Recycled filament can be produced from post-industrial or post-consumer ABS waste, for example, discarded automotive or motorcycle components/electronic housings and defective 3D prints. Technical recommendations for achieving optimal results with rABS are provided in Table 2. A comparison of selected mechanical property characteristics of recycled/virgin ABS is shown in Figure 3. In terms of rheological properties, no significant variations in the melt flow index (MFI) are reported between the first and fifth recycling cycles, with values ranging from 1.44 to 1.51 g/10 min. This indicates that only minimal changes in the viscosity of ABS occur over the examined recycling cycles [18,19,20,21,22].

### 3.2. Recyclate Polycarbonate

**Figure 4:**
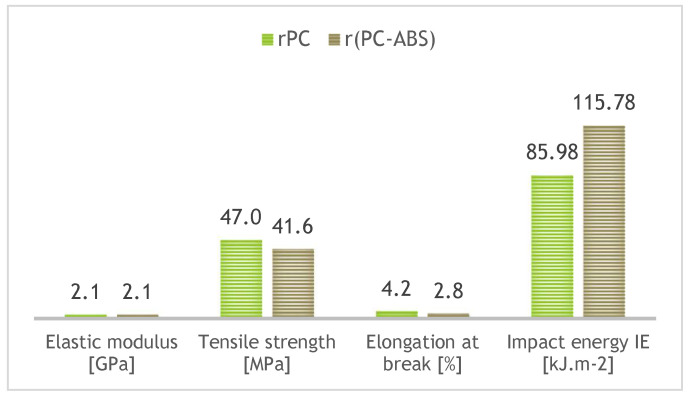
Comparison of selected mechanical property characteristics of rPC and recycled PC-ABS blend (mechanical properties—tensile and impact following ASTM D638 and D6110-18) [23].

PC is one of the most widely used engineering (amorphous) thermoplastics, with applications in the automotive, pharmaceutical, and construction industries. Polycarbonates exhibit an extended ductile region and a fairly high fracture strain; they are considered hard, tough materials. The material is transparent, tough, thermally/dimensionally stable, with a density of 1.2 g·cm^−3^ (and can maintain rigidity up to 140 °C and toughness down to −20 °C). It can also be produced in a bio-based form by copolymerization of limonene oxide (derived from limonene, which is found in orange peels) and CO_2_. PC-ABS blends offer a higher heat distortion temperature (compared with ABS), with good processing characteristics. Typical unfilled PC-ABS blends have a tensile modulus in the range of 2000–2300 MPa. The possibilities of using recyclate for 3D printing filament production are reported in studies investigating reductions in mechanical property values, lower thermal stability, and weakening of interfacial adhesion. A comparison of rPC and recycled PC-ABS blend is addressed in study [23]. In general, the application of the recycled blend achieved better printability while maintaining the required dimensional accuracy. A comparison of selected mechanical property characteristics is shown in Figure 4 [16,17,23,24,25,26,27,28].

The assessment of rheological property indicators within the aforementioned studies is mentioned by the authors of [27]. The melt flow index (MFI) of filament produced from recycled material is reported to be approximately 35% higher compared to that of virgin polymer.

### 3.3. Recyclate Polyamide/Nylon

The term nylon (PA—polyamide) refers to a group of synthetic polymers characterized by the presence of amide groups (CO-NH) in the main polymer chain (engineering polymers). The numbers following the polyamide designation (PA6, PA6.6, PA12, etc.) characterize the precursor monomers according to the number of carbon atoms in their molecules. Application areas of nylon include packaging and consumer goods (textile fibers, fishing nets), automotive equipment/engines/tires, and electronics and electrical components [14,29].

In general, polyamides are tough materials with relatively high tensile strength and good resistance to abrasion, wear, heat, and chemicals. By varying monomers, it is possible to make products that are hard and tough or soft and rubbery. However, nylon is susceptible to degradation processes induced by moisture absorption and ultraviolet (UV) radiation. Prolonged exposure to these factors results in the deterioration of mechanical properties [30].

**Figure 5:**
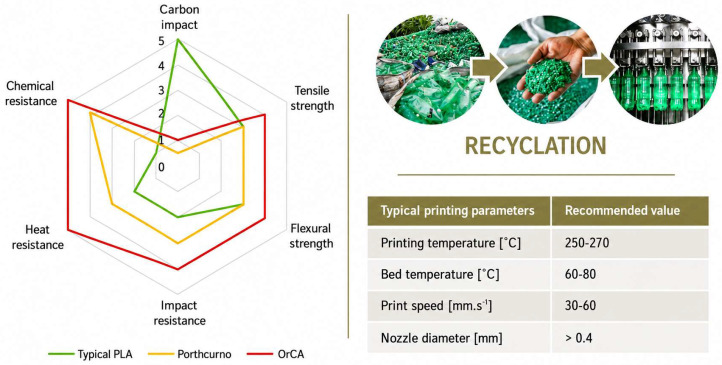
Comparison of properties: typical PLA versus recyclates developed by FishyFilaments; on the right, the recycling process is shown (material supplied by Reflow and typical printing parameters PORTHCURNO). Printing conditions—recommended by the filament supplier [31,32].

Differences in the properties of PA6, PA6.6, and PA12 are presented in Table 3. Recycled nylon (also referred to as Econyl) is most commonly produced from textiles, fabrics, fishing nets, etc. The actual percentage of produced recyclate accounts for only 2% of total PA waste. Companies such as Aquafil and FishyFilaments offer multiple products (filaments) in their portfolios manufactured from nylon monofilament by recycling fishing nets. A comparison of the properties of standard Polylactic acid (PLA) versus recyclates is shown in Figure 5 [31,32,33].

Study [34] reports that the physical and mechanical properties of recycled nylon (PA6) are practically identical to those of virgin plastic. The number of cycles without significant changes in properties is three. Study [35] indicates that the optimal number of recycling cycles is one to two (production technology—injection molding). To improve the thermal resistance of the filament strand, a filler may be applied, e.g., recycled PA6 + Al_2_O_3_ filler. Changes in properties are also observed in specimens produced with only a certain proportion of recyclate [34,35,36,37].

### 3.4. Recyclate Acrylonitrile Styrene Acrylate

Acrylonitrile styrene acrylate (ASA) is a tough, rigid material resistant to impact (even at low temperatures) and chemicals, and thermally stable, making it suitable for outdoor applications, automobiles, electronics, aerospace, marine applications, etc. Structurally similar to ABS, it was developed as an alternative with increased resistance to weathering and ultraviolet radiation; however, it is sensitive to residual moisture. This is due to the grafting of an acrylic ester elastomer onto the styrene-acrylonitrile backbone. Its *T_g_* is lower than that of ABS, which contributes to its superior low-temperature properties. Printing conditions are similar to those for ABS. ASA is recyclable (marked with recycling symbol 7—Other); however, only a limited number of companies actually have equipment for sorting and chipping. Therefore, the material is often energy-recovered by incineration, as it has a high calorific value. Within EU countries, the material is marketed under the trade name Luran^®^S (manufacturer: the German group BASF SE). The number of publications on ASA recycling is limited. One available study is [38], in which the authors compare the thermomechanical properties of recyclates after individual recycling cycles (1–6 cycles). The differences in mechanical properties of recycled filaments are presented in Table 4 [16,38,39,40,41].

rASA filament is more prone to the formation of voids and pores; the phenomenon is more pronounced when expansive additives are present in the recyclate. Optimization of FDM printing parameters for parts manufactured from rASA (and rPETG) is addressed in study. Based on the obtained results of mechanical property tests (tensile strength, compression) and calculated cost, the specimen with layer height = 0.20 mm/density percentage = 100% was identified as optimal [42,43].

### 3.5. Recyclate Polyethylene Terephthalate

PET is a polycondensate of terephthalic acid and ethylene glycol, patented in 1941 (classified as a polyester). Depending on processing conditions, it can range from semi-rigid to rigid. PET is hard, stiff, and strong, with good dimensional stability, a favorable strength-to-weight ratio, chemical resistance (except to alkalis), excellent electrical insulating properties, and is practically shatter-resistant (making it suitable as a glass substitute). The glass transition temperature of PET varies depending on the degree of crystallinity. Commercial applications include more than 85% of PET for fiber production, as an injection-molding-grade material, for blow-molded gas-tight bottles for carbonated beverages, and for oriented films. Two methods are used for PET recycling: direct reuse after comminution and washing, or alcoholysis. In the case of foils and films, chemical recycling (pyrolysis) is often the only option due to composition (in most cases, composite materials). The effect of multiple recycling cycles on mechanical performance includes a decrease in tensile strength (by 7.5 MPa) and elongation at break (by ~37%) in the third recycling cycle (vPET). The addition of glycol increases the number of possible recycling cycles [25,39,44,45,46].

Studies on the production of filament from single-use PET bottles over the past five years are analyzed in Table 5.

### 3.6. Recyclate Polypropylene

PP is characterized by low density (0.904–0.908 g·cm^−3^), high mechanical strength, toughness, and chemical resistance. It is electrically insulating and, upon contact with moisture and liquids, exhibits minimal water absorption. Its application is limited by a high thermal expansion coefficient. It is used in the production of disposable food packaging and bottles, which contributes to a large amount of post-consumer waste. For separation of PET and PP bottles, the sink/float method can be used based on polymer specific gravity. At present, PP recycling is not as economically viable as that of other polymers due to difficulties in collection, possible contamination, and mixing with other materials [16,58,59,60,61].

In general, it is reported that closed-loop recycling of PP is possible approximately four times before significant thermal degradation of the polymer occurs. In many cases, rPP filaments are blended with virgin polymer at a 30/70 wt.% ratio, or the recyclate is combined with natural reinforcements for property modification, e.g., rice husk, harakeke, hemp fiber, recycled gypsum, etc. Adding an appropriate percentage of fibers increases filament strength characteristics (tensile strength, Young’s modulus) and reduces shrinkage of 3D prints. A disadvantage is possible moisture absorption and swelling due to the natural hydrophilicity of the fibers. PP can be recycled into many different products, including electrical and electronic equipment. Recycled filament produced from this type of waste exhibits better rheological properties due to the presence of talc. The decomposition temperature of recycled filament is lower than that of HDPE, and fracture surfaces appear more brittle. Recycled PP is, however, still not widely used for 3D printing filament production due to warping and poor interlayer adhesion issues [61,62,63,64,65,66,67,68].

### 3.7. Recyclate High-Density Polyethylene

A HDPE is relatively lightweight and has a high strength-to-density ratio. It is flexible, translucent/waxy, moisture-resistant, shows good chemical resistance and impermeability, and has excellent electrical insulating properties. It is used in packaging applications, decorative textiles, automotive components, and pipes/fittings. HDPE was long considered the second most recyclable plastic (but overtook PET several years ago). A major early market for recycled HDPE was agricultural drainage pipe (2003, USA). The HDPE recycling process is adversely affected by fillers and colorants. From an economic perspective, filament production is more efficient from recyclate than from virgin polymer—the cost is several times lower. rHDPE exhibits optimal properties between the second and fifth recycling cycles; in the sixth cycle, impact toughness and tensile strength decrease. The optimal printing temperature is approximately 230 °C while maintaining the required print characteristics. The melt flow index (MFI) of recycled materials is reported to be higher compared to that of virgin polymers (by approximately 4%—after the first recycle cycle). This increase is attributed to polymer chain scission induced by repeated processing and remelting of the recyclate. An elevated MFI is considered to have a positive effect on interlayer coalescence between printed layers. Some studies report limited applicability of rHDPE in functional or load-bearing 3D-printed components due to inconsistent filament geometry, poor adhesion, and persistent deformation problems—warping/shrinkage [16,59,69,70,71,72].

### 3.8. Recyclate Poly Methyl Methacrylate

Advantages of poly methyl methacrylate (PMMA) include dimensional stability, resistance to moisture/scratching/impact, certain chemical resistance, and good machinability. It is a brittle, transparent material of moderate mechanical strength, and its heat resistance is insufficient for higher-temperature use, as the service temperature is only about 80 °C. It is used in the automotive industry (lightweight and aesthetic), construction (durability, UV resistance), medical devices/permanent 3D implants (biocompatibility, easy sterilization), and optics (favorable refractive index, excellent UV resistance). PMMA is marked with recycling code 7. It can be recycled chemically (pyrolysis) or mechanically. Mechanical recycling is problematic due to industrial additives and chemicals applied. Under recycling-center conditions, it is cost-ineffective or impracticable—even with effective separation. PMMA recycling is expected to reduce energy consumption, volatile organic compound (VOC) emissions, and CO_2_ emissions by at least 66%, 60%, and 50%, respectively. The tensile strength of rPMMA remains comparable to virgin PMMA up to the fourth recycling cycle, while a decrease occurs in the sixth cycle. The sixth recycling cycle is also a turning point in rheological properties, with the melt flow index (MFI) value decreasing to one-third of the value in the first recycling cycle. The cost of recycled filament production was estimated by rough calculation at approximately 42% of the cost of virgin PMMA [16,73,74,75].

### 3.9. Recyclate Polyether Ether Ketone and Polyetherimide

**Figure 6:**
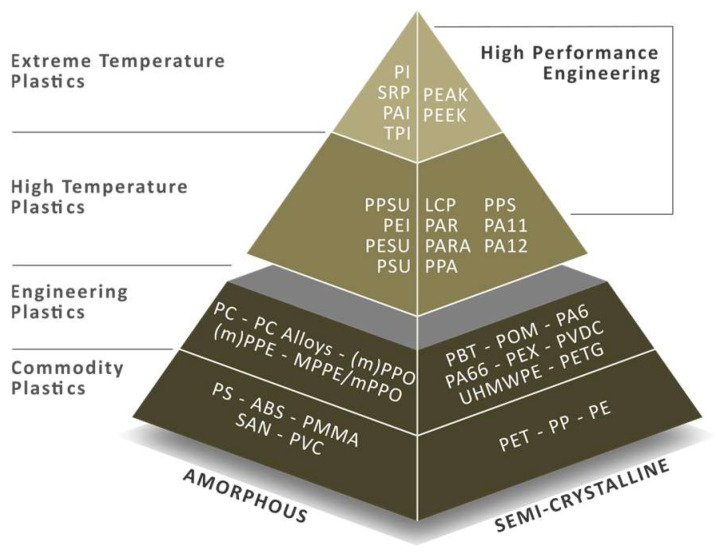
Classification of plastics based on properties and application areas (PEEK positioned at the “top of the pyramid”).

Polyether ether ketone (PEEK) is a highly resistant and rigid, yet flexible polymer, originally developed primarily for applications in the manufacture of aircraft components, piston parts, cable insulation, and turbine blades. Owing to its specific properties (biocompatibility, osseointegration, aesthetics, morphology, radiolucency, etc.), it is also suitable for healthcare/pharmaceutical applications. It belongs to the group of high-performance (see Figure 6) fully recyclable plastics, produced in low tonnages due to cost. 3D printing of PEEK products has attracted intensive interest over the last decade, but only a very small percentage of studies focus on filament production from recycled PEEK. Experimental recycling of PEEK/carbon fiber composites is also reported in [76]. Where the recyclate exhibits reduced tensile/shear strength to approximately 40% of the original level. In such cases, a lower-value material is obtained, i.e., so-called downcycling. PEEK can be recycled mechanically as well as chemically. Mechanical recycling is the more suitable alternative due to:

- Preservation of molecular integrity;
- Minimal energy consumption;
- Zero chemical waste (compared with solvent-, or pyrolysis-based methods).

Polyetherimide (PEI), similarly to PEEK, is classified as a high-performance engineering plastic. Recycling of these types of plastics is more challenging compared to commodity/engineering plastics (it is not recycled in conventional commercial plants). Its recycling is carried out by specialized companies [76,77,78,79,80,81].

### 3.10. Description of Degradation Mechanisms in the Process of Mechanical Recycling

Repeated thermal loading leads to so-called degradation processes. Polymer degradation can be described as a complex alteration of the chemical structure and physical properties of the material. This process is driven by a combination of elevated temperatures and pressures, shear forces, and the presence of a limited amount of oxygen dissolved in the polymer matrix [81,82].

The individual factors contribute as follows [83,84,85,86,87,88,89,90]:

- Temperature → thermal degradation;
  ○ Mechanisms of thermal degradation include:
  ○ (1) Depolymerization—typical for PMMA; the decrease in the degree of polymerization is initially negligible, and consequently the mechanical properties do not deteriorate rapidly. (2) Random chain scission—chain rupture occurs at random points along the polymer backbone, leading to a noticeable reduction in molecular weight (*M_w_*) and a corresponding rapid deterioration of mechanical properties, typical for polymers such as PP and PE. (3) Degradation via substituent reactions—involving modification or elimination of substituent groups attached to the polymer backbone. Mechanisms (1) and (2) may occur independently or simultaneously.
- Temperature/mechanical stresses → thermal-mechanical degradation;
  ○ Polymers are exposed to thermo-mechanical degradation within the confined flow of the extruder, where oxygen access is limited. In addition to heat, the material is subjected to shear forces. The most common mechanisms in commercial polymers are chain scission and chain branching, depending on the polymer type, *M_w_*, and processing temperature. A typical example of chain scission is PP, whereas certain types of PE tend to exhibit chain branching. Changes in *M_w_* significantly affect rheological properties and mechanical behavior; for instance, a decrease in *M_w_* in PET results in reduced elongation. Thermo-mechanical degradation directly affects elongation and impact resistance.
- Temperature/oxygen → thermal-oxidative degradation;
  ○ This degradation mechanism occurs due to the combined effect of heat and oxygen. The main polymer chain bonds are progressively cleaved, resulting in a decrease in *M_w_*. The degradation pathway is influenced by structural heterogeneities and the weakest points in the polymer chain, which act as initiation sites for degradation reactions.
- Moisture → hydrolysis;
  ○ Hydrolysis is caused by excess moisture; reactions with H_2_O lead to cleavage of chemical bonds and a reduction in *M_w_*. Moisture-sensitive polymers include polyesters, polyamides (PA), and polycarbonates (PC); therefore, pre-drying of the granulate prior to extrusion is required.

## 4. Discussion

The information presented in Section 3.1, Section 3.2, Section 3.3, Section 3.4, Section 3.5, Section 3.6, Section 3.7, Section 3.8 and Section 3.9 was synthesized through a systematic keyword-based search, including terms related to specific polymers in recycled form (e.g., rABS, rPP, rPET) and their application in filament production. The primary selection criterion for source documents was their scientific rigor and thematic relevance to the investigated topic. Based on this synthesis, a systematic comparison of the results reported in individual studies was conducted using common evaluation criteria—mechanical properties (number of recycling cycles), rheological characteristics (MFI), and printability. Based on these findings, the materials can be compared as follows.

1. Mechanical properties (recycling cycles):
  - rABS—most studies report minimal changes compared to virgin ABS (vABS), particularly at lower numbers of recycling cycles; in blends, the properties of the recyclate tend to stabilize (approximately up to 5 cycles without significant degradation);
  - rPC/PC-ABS—in the case of recycled polycarbonate (rPC), a decline in mechanical properties is observed; PC-ABS blends represent a compromise between performance and processability;
  - rPA—properties are nearly identical to those of the virgin material at lower numbers of recycling cycles; thermal stability can be improved by the addition of Al_2_O_3_ (typically after 1–3 recycling cycles);
  - rASA—repeated recyclability is reported up to approximately 3–5 cycles, at around 6 cycles, a significant change in viscosity is observed;
  - rPET—the third recycling cycle is critical due to the significant decrease in elongation at break;
  - rPP/rHDPE—rPP can typically be processed up to approximately 4 cycles (with property modification achievable through the addition of natural fillers); for rHDPE, maintaining optimal properties is generally feasible within 2–5 recycling cycles;
  - rPMMA—mechanical properties remain relatively stable up to approximately 4 cycles, followed by a noticeable decline;
  - rPEEK—preservation of complete structural integrity over 3 recycling cycles.
2. Rheological properties—MFI (viscosity):
  - rABS—MFI shows minimal variation with increasing number of recycling cycles (1.44–1.51 g/10 min between cycles 1 and 5) → indicating stable processability;
  - rPC—an increase in MFI of approximately 35% is reported (after the first recycle process);
  - rASA—significant increase in MFI of approximately 98% is observed at the sixth recycling;
  - rPP/rHDPE—rheological properties of rPP improve in the presence of talc in recycled products; for rHDPE, an increase in MFI of approximately 4% is observed after the first-repeated thermal processing;
  - rPMMA—MFI decreases significantly at the sixth recycling cycle, reaching approximately one-third of its original value.
3. Printability (technological property):
  - rABS—good printability; however, deformation must be controlled;
  - PC-ABS—good printability;
  - rPET/rPA—sensitive to moisture; pre-processing drying is required;
  - rPP/rHDPE—susceptible to deformation and poor adhesion;
  - rASA—tendency for pores and voids formation.

Based on the above evaluation, rABS emerges as the most balanced material for 3D printing applications. In contrast, rPP and rHDPE can be considered problematic materials due to technological limitations associated with their processing in additive manufacturing. High-performance polymers represent a particular challenge in secondary processing, primarily due to relatively low production volumes, specific material properties, and the insufficiently developed recycling infrastructure for their efficient collection, sorting, and reprocessing (in comparison with commodity and engineering plastics).

Given the current relevance of this topic, several research gaps can be identified. These include, in particular, the lack of a unified methodology for evaluating the properties of recycled materials, as well as the absence of reliable predictive models capable of estimating material behavior after multiple recycling cycles. At the same time, the negative impacts of mechanical recycling—such as the generation of microplastics—remain insufficiently investigated, along with the influence of various additives and modifiers on the properties of materials processed into recyclates.

## 5. Conclusions

There are multiple reasons for the application of recycled materials in 3D printing, ranging from environmental benefits and reduced production costs to localized manufacturing and the promotion of sustainable technologies and circular economy (CE) principles. Recycled polymers, even after repeated thermal processing, can retain mechanical and rheological properties comparable to those of virgin materials. However, progressive degradation of polymer chains inevitably occurs, leading to a reduction in strength and toughness, as well as changes in melt viscosity. The maximum number of feasible recycling cycles varies depending on the type of polymer. When selecting filament material, virgin versus recycled, the intended function of the printed part must be carefully considered. If the printed component serves as a load-bearing element or is subjected to long-term or extreme loading conditions, the use of recycled material must be critically evaluated. Recycling, as a technological process of waste reprocessing, represents only one of the strategies within the circular economy framework. A systematic evaluation of recyclability should be integrated already at the plastic product design stage, with a strong emphasis on eco-design principles. At the same time, it is essential to encourage end users to minimize excessive plastic waste, recognizing that no plastic material can be recycled indefinitely. The identified research gaps represent open challenges and define directions for future research in this field.

## Figures and Tables

**Table 1:** Comparison of global plastics production (2020–2024) on different bases, values in million tons [9].

Type/Year	2020	2021	2022	2023	2024
Fossil-based	347.3	359.8	362.3	374.2	387.0
Mechanically recycled (post-consumer)	31.6	32.5	35.5	36.2	40.8
Bio-based (including bio-attributed since 2022)	1.5	1.7	2.3	3.0	2.6
Chemically recycled (post-consumer)	0.2	0.2	0.2	0.3	0.4
Carbon-captured	-	-	0.1	0.1	0.1

**Table 2:** Technical recommendation for achieving optimal results from rABS [20].

Parameter	Recommended Value	Notes
Nozzle temperature [°C]	230–260	Adjust based on printer and filament brand
Bed temperature [°C]	90–110	Essential for adhesion
Heated bed	Required	Prevents warping
Enclosure	Recommended	Minimize drafts and cooling issues
Print speed [mm·s^−1^]	40–60	Balanced speed and quality
Cooling fan	Off or minimal	Reduces layer separation
Ventilation	Well-ventilated area	Use enclosure with filtration if available
Storage	Airtight, dry container	Prevents moisture absorption

**Table 3:** Properties of unmodified polyamides (PA6, PA6.6, and PA12—dry form and 50% R. H.—relative humidity) [25].

Property	PA6 Dry (50% R. H.) ^1^	PA6.6 Dry (50% R. H.) ^1^	PA12 Dry (50% R. H.) ^1^
Tensile strength [MPa] (ASTM D638)	81 (69)	83 (77)	49 (47)
Ultimate elongation [%] (ASTM D638)	200 (300)	60 (≥300)	250 (250)
Flexural modulus [MPa] (ASTM D790)	2700 (900)	2830 (1210)	1410 (1030)
Izod impact strength [J·m^−1^] (ASTM D256)	58 (215)	53 (112)	58 (64)

^1^ Water absorption after 24 h (50% R. H.).

**Table 4:** Mechanical and rheological properties of recycled filaments [38].

Characteristics	Value (1st Recycling Cycle)	Percentage Increase/ the Highest Achieved Value
Tensile strength [MPa] (ASTM D638)	39.6	+20.0%/max. value 47.5 (2nd recycling cycle)
Tensile modulus of elasticity [MPa] (ASTM D638)	146.4	+37.3%/max. value 201 (2nd recycling cycle)
Flexural strength [MPa] (ASTM D790)	73.6	+24.5%/max. value 91.6 (4th recycling cycle)
Flexural modulus of elasticity [GPa] (ASTM D790)	2.09	+26.7%/max. value 2.64 (4th recycling cycle)
MFI [g/10 min] (ASTM D1238-13)	8.86	+98.8%/max. value 17.61 (6th recycling cycle)

**Table 5:** Areas of study within PET recycling [47,48,49,50,51,52,53,54,55,56,57].

Area of Interest	Recommendations (Directions for Further Study)/References
Ovality/filament diameter in extrusion from rPET. MFI and viscosity of vPET/rPET/vPETG (Polyethylene terephthalate glycol-modified) with different moisture contents. Thermogravimetric, DSC analysis, mechanical properties.	The material is moisture-sensitive, ensures effective moisture removal. Seibert et al. (2022) [47]
Chemical/thermal characterization (molar mass, *T_g_*, *T_cc_*, *T_m_*_,1_, *T_m_*_,2_, etc.) Optimal extrusion parameters and mechanical properties. 3D printing—temperature, shrinkage, effect of printing direction on mechanical properties, and effect of build orientation on cohesion between tracks.	– Van De Voorde et al. (2022) [48]
Extrusion/degradation temperature (set depending on the PET grade). Mechanical properties of rPET/vPET → comparable. Only a limited effect of crystallinity on the material was observed due to thermal processing, as evidenced by XRD.	– Kapil Ror et al. (2023) [49]
Recycling PET bottles, sustainability. Mechanical properties of rPET/PLA → comparable.	– Nikam et al. (2023) [50]
Modification of rPET filament by the addition of EBA-GMA. Study of physical properties and thermal characterization (*T_g_*) + SEM micrographs of fractures after the tests.	– Toth et al. (2024) [51]
Effect of nozzle temperature, print speed, infill density, and layer thickness of 3D-printed rPET specimens on their strength characteristics.	(Degradation of material properties after multiple recycling cycles). O’Driscoll et al. (2024) [52]
Comparison of the strength characteristics of filament.	Includes remarks on further testing of samples. Pepek & Hanan (2025) [53]
The effect of different printing temperatures on the physio-mechanical properties of 3D-printed samples produced from vPET and rPET filament (recyclate form—pellets, flakes).	– Mishra et al. (2025) [54]
The crystallinity and *T_g_* of rPET and vPET filaments. The need for a circular economy was emphasized.	– Amor et al. (2025) [55]
Mechanical testing of specimens manufactured from rPET, PLA, and PETG.	The use of rPET is recommended for low-load prototypes and for applications in academic environments. Alyr et al. (2025) [56]
Surface roughness parameters of turned workpieces manufactured by 3D printing from rPET modified by the addition of EBA-GMA (0–20 wt%).	(The effect of the modifier on the base material in relation to machining). Kónya et al. (2025) [57]

## Data Availability

No new data were created or analyzed in this study.

## Abbreviations

The following abbreviations are used in this manuscript:

ABS	Acrylonitrile butadiene styrene
ASA	Acrylonitrile styrene acrylate
CE	Circular economy
EBA-GMA	Ethylene butyl acrylate glycidyl methacrylate
EU	European union
FDM	Fused deposition modeling
HDPE	High-density polyethylene
LDPE	Low-density polyethylene
MFI	Melt flow index
*M_w_*	Molecular weight
PA	Polyamide
PC	Polycarbonate
PEI	Polyetherimide
PEEK	Polyether ether ketone
PET	Polyethylene terephthalate
PETG	Polyethylene terephthalate glycol-modified
PLA	Polylactic acid
PMMA	Poly methyl methacrylate
PP	Polypropylene
PS	Polystyrene
PVC	Polyvinylchloride
RH	Relative humidity
*T_g_*	Glass transition temperature
UV	Ultraviolet
VOC	Volatile organic compound
